# ﻿*Primulinananlingensis* (Gesneriaceae), a new species from the Limestone Karst of Guangdong, China

**DOI:** 10.3897/phytokeys.254.145138

**Published:** 2025-03-26

**Authors:** Jin-Chu Luo, Yuan-Qiu Li, Ya-Li Li, Ming-Zhao She, Yang-Jin Zeng, Fa-Guo Wang, Hong-Feng Chen

**Affiliations:** 1 Guangdong Provincial Key Laboratory of Applied Botany, State Key Laboratory of Plant Diversity and Specialty Crops, South China Botanical Garden, Chinese Academy of Sciences, Guangzhou 510650, China South China Botanical Garden, Chinese Academy of Sciences Guangzhou China; 2 University of Chinese Academy of Sciences, Beijing 100049, China University of Chinese Academy of Sciences Beijing China; 3 Guangdong Shimentai National Nature Reserve, Yingde 511500, China Guangdong Shimentai National Nature Reserve Yingde China

**Keywords:** Gesneriaceae, limestone flora, morphology, phylogeny, taxonomy

## Abstract

*Primulinananlingensis*, a new species of Gesneriaceae from the Karst of Guangdong, China, is described and illustrated. This species is morphologically similar to *P.versicolor*, but can be distinguished by its larger crenate-margined leaves, fewer flowers per cyme and overall cyme number, ovate-lanceolate bracts with shallow serrations, calyx lobes with 1–3 teeth per side, stamens densely glandular at base and tip and pistil densely glandular-puberulent. It also resembles *P.pengii*, but has significant differences in its longer corolla, pale yellow corolla colour and ovate-lanceolate bracts. Phylogenetic analyses with ITS and *trn*L-F sequences revealed that *P.nanlingensis* is sister to *P.versicolor* and *P.pengii*, but isolated from its morphological relatives. The phylogenetic and morphological relationships with similar species are discussed, including detailed descriptions, photographs and distribution information. According to the IUCN Red List Criteria, the new species is assessed as Near Threatened [NT].

## ﻿Introduction

The genus *Primulina*Hance (1883) was initially monotypic, with its type specimen collected from the Lianjiang River Basin in northern Guangdong, China. The second species, *P.guangxiensis* Yan Liu & W.B.Xu, was described in 2011, based solely on morphological characteristics (Liu et al. 2011). Drawing upon molecular and morphological evidence, *Primulina* underwent subsequent revision and expansion, resulting in the identification of 123 species and eight varieties (Wang et al. 2011; Weber et al. 2011). The revision included numerous species from Chiritasect.Gibbosaccus, two species from *Wentsaiboea* D.Fang & D.H.Qin and *Chiritopsis* W.T.Wang which were consolidated into *Primulina* (Zhou et al. 2016; Xu et al. 2023). Species within the genus *Primulina* are perennial herbs distinguished by their rhizomatous stems, fleshy opposite leaves, corolla with an infundibuliform tube and 2-lipped lobe, two stamens with coherent anthers and a characteristic chiritoid stigma (Wang et al. 2011; Yang et al. 2023). As of February 2025, the genus *Primulina* has 244 accepted species, thereby constituting the largest genus in the Chinese Gesneriaceae (Chen et al. 2024; GRC 2025).

Typically residing in the sheltered and moist conditions of karst formations, such as caves and similar microhabitats (Xu et al. 2021), these species are predominantly restricted to limestone niches in southern and south-western China, as well as northern Vietnam (Kang et al. 2014; Li et al. 2019). Globally, the genus includes over 170 species that are endemic to karst regions (Xu et al. 2021; Pan et al. 2022). Guangdong Province is home to 114 species of Gesneriaceae, with a significant proportion of 48 species belonging to *Primulina* (Song et al. 2023). Since 2017, four new species of *Primulina* have been described in Guangdong Province, these being *P.effusa* F.Wen & B.Pan, *P.anisocymosa* F.Wen, Xin Hong & Z.J.Qiu, *P.huangjiniana* W.B.Liao, Q.Fan & C.Y.Huang and *P.liangwaniae* B.M.Wang & Y.H.Tong (Pan et al. 2017; Hong et al. 2019; Huang et al. 2020; Tong et al. 2023).

During a botanical survey conducted in 2024 within the Shimentai Nature Reserve, an unidentified species of *Primulina* was discovered on two limestone hills. The species was subsequently introduced and cultivated in the greenhouses of the South China Botanical Garden, Chinese Academy of Sciences. From April to June of 2024, the plant exhibited continuous flowering with beautiful pale yellow flowers. After rigorous comparison of this material with herbarium specimens and consultation of relevant references and monographs (Guo et al. 2015; Pan et al. 2016), we confirmed that it represents a new species of *Primulina*, which we describe in this study. Phylogenetic analysis, utilising ITS and *trn*L-F sequences, confirmed its position within the genus.

## ﻿Material and methods

### ﻿Morphological observation

The material of this new species was collected during a botanical survey conducted at Shimentai Nature Reserve, Yingde City, Guangdong Province. The species was cultivated for further morphological study at the South China Botanical Garden, Chinese Academy of Sciences. Morphological assessments of the new species were carried out using herbarium specimens, with measurements taken from fresh samples. Comparative morphology was conducted with morphologically similar species, utilising both living plants and specimens from institutions such as IBSC, KUN, PE and IBK, as well as digital images from JSTOR Global Plants (http://plants.jstor.org/). Indumentum characteristics were examined by an Olympus-SZ61 stereomicroscope and Olympus-BX43 optical microscope, with photographic documentation accomplished using a Nikon D810 camera.

### ﻿Molecular sampling

We collected one individual from each of the two natural populations within the protected area and dried the fresh leaf samples using silica gel. Genomic DNA was extracted from the dried leaves using a modified CTAB protocol (Doyle and Doyle 1987). Based on recent phylogenetic studies (Pan et al. 2022; Yang et al. 2023; Chen et al. 2024), we retrieved ITS and *trn*L-F sequences for 124 *Primulina* species from GenBank to determine the phylogenetic position of the new species. *Petrocodonainsliifolius* W.H.Chen & Y.M.Shui and *Petrocodonhancei* (Hemsl.) A.Weber & Mich.Möller were selected as outgroup taxa for the analysis (Chen et al. 2024). Corresponding GenBank accession numbers are presented in Table 1.

**Table 1.** Species names and GenBank accession numbers of ITS and *trn*L-F DNA sequences used for analysis.

Species	ITS	*trn*L-F	Species	ITS	*trn*L-F
*Petrocodonainsliifolius*	KF202291	KF202298	Primulinalunglinensisvar.amblyosepala	MK747105	MK746281
*Petrocodonhancei*	KY796057	KY796059	*Primulinalungzhouensis*	KY394931	KY393525
*Primulinaalutacea*	KY394847	KY393441	*Primulinalutea*	JX506921	JX506813
*Primulinaargentea*	KY394848	KY393442	*Primulinalutvittata*	MK369978	MK369993
*Primulinabaishouensis*	KY394849	KY393443	*Primulinamabaensis*	KY394937	KY393531
*Primulinabalansae*	MK747141	MK746274	*Primulinamacrodonta*	JX506923	JX506815
*Primulinabeiliuensis*	KY394850	KY393444	*Primulinamaculata*	KU220604	KU220609
Primulinabeiliuensisvar.fimbribracteata	KY394851	KY393445	*Primulinamalipoensis*	MK747123	MK746240
*Primulinabicolor*	KY394852	KY393446	*Primulinamedica*	KY394940	KY393534
*Primulinabipinnatifida*	KY394853	KY393447	*Primulinamelanofilamenta*	MK747158	MK746277
Primulinabrachytrichavar.magnibracteata	MK369979	MK369994	*Primulinaminor*	MK747160	MK746290
*Primulinacarinata*	KY394858	KY393452	*Primulinaminutimaculata*	KY394941	KY393535
*Primulinacataractarum*	MW900263	MW960358	*Primulinamoi*	KF498115	KY393536
*Primulinachizhouensis*	KY394860	KY393454	*Primulinamollifolia*	KY394943	KY393537
*Primulinaconfertiflora*	MK747101	MK746253	*Primulinanandanensis*	KY394947.1	KY393541
*Primulinacordata*	KC190200	KC190207	*Primulina nanlingensis_*YD1	PQ740297	PQ759014
*Primulinacordistigma*	MK747118	MK746251	*Primulina nanlingensis_*YD2	PQ740298	PQ759015
*Primulinacrassirhizoma*	KY394864	KY393458	*Primulinaningmingensis*	KY394949	KY393543
*Primulinacrassituba*	MK747147	MK746230	*Primulinaobtusidentata*	KF498096	KY393544
*Primulinacurvituba*	MK747137	MK746242	*Primulinaophiopogoides*	KF498062	KY393545
*Primulinadanxiaensis*	JX506886	JX506778	*Primulinaorthandra*	MK747128	MK746286
*Primulinadepressa*	KY394869	KY393463	*Primulinapengii*	KU220603	KU220610
*Primulinadiffusa*	KY394871	KY393465	*Primulinapetrocosmeoides*	KY394953	KY393547
*Primulinadongguanica*	KY394872	KY393466	*Primulinapinnatifida*	KY394954	KY393548
*Primulinadryas*	KY394875	KY393469	*Primulinapolycephala*	KY394955	KY393549
*Primulinaeburnea*	JX506891	JX506783	*Primulinaporphyrea*	KU173793	KU173799
*Primulinaefusa*	MK369976	MK369991	*Primulinapseudoeburnea*	KY394958	KY393552
*Primulinafengkaiensis*	MK369975	MK369990	*Primulinapseudoglandulosa*	KF498138	KY393482
*Primulinafengshanensis*	MK369970	MK369985	*Primulinapseudoheterotricha*	JX506933	JX506824
*Primulinafimbrisepala*	JX506894	JX506786	*Primulinapseudolinearifolia*	MK747140	MK746280
Primulinafimbrisepalavar.mollis	JX506895	JX506787	*Primulinapseudoroseoalba*	KY394959	KY393553
*Primulinafordii*	MG727881	MG727878	*Primulinapungentisepala*	KY394962	KY393556
Primulinafordiivar.dolichotricha	MK747125	MK746247	*Primulinapurpurea*	KY394964	KY393558
*Primulinaglandaceistriata*	MK747114	MK746256	*Primulinaqingyuanensis*	KY394965	KY393559.1
*Primulinaglandulosa*	KY394887	KY393481	*Primulinarenifolia*	KY394966	KY393560
*Primulinagongchengensis*	KY394889	KY393483	*Primulinaroseoalba*	KY394972	KY393566
*Primulinagrandibracteata*	MK747121	MK746266	*Primulinarosulata*	KU528874	KU528884
*Primulinaguihaiensis*	KY394893	KY393487	*Primulinarubribracteata*	KU173791	KU173797
*Primulinahalongensis*	KY394895	KY393489	*Primulinasecundiflora*	MK747119	MK746279
*Primulinahedyotidea*	JX506905	JX506797	*Primulinashouchengensis*	KY394980	KY393574
*Primulinaheterochroa*	KY394898	KY393492	*Primulinasichuanensis*	MK747162	MK746264
*Primulinahochiensis*	JX506903	JX506795	*Primulinasinovietnamica*	MK369973	MK369988
*Primulinahuaijiensis*	KF498127	KY393495	*Primulinaspinulosa*	KF498063	KY393576
*Primulinahunanensis*	KU220602	KU220608	*Primulinasubulata*	KY395020	KY393579
*Primulinajiangyongensis*	KY394902	KY393496	Primulinasubulatavar.guilinensis	KY394967	KY393561
*Primulinajingxiensis*	KY394903	KY393497	*Primulinasubulatisepala*	MK747122	MK746246
*Primulinajiulianshanensis*	OP243287	OP243283	*Primulinasuichuanensis*	KY395021	KY393580
*Primulinajiuwanshanica*	MK747116	MK746260	*Primulinatabacum*	KY395023	KY393582
*Primulinajuliae*	MG727889	MG727873	*Primulinatenuituba*	KY395025	KY393584
*Primulinalangshanica*	KY394907	KY393501	*Primulinatsoongii*	KY395029	KY393588
*Primulinalatinervis*	KY394908	KY393502	*Primulinaverecunda*	KY395031	KY393590
*Primulinalechangensis*	KY394910	KY393504	*Primulinaversicolor*	MK747155	MK746252
*Primulinaleeii*	KY394911	KY393505	*Primulinavestita*	MK747156	MK746282
*Primulinalepingensis*	KY394913	KY393507.1	*Primulinavillosissima*	KY395032	KY393591
*Primulinalianpingensis*	MH343910	MH344542	*Primulinawenii*	MK747148	MK746284
*Primulinalijiangensis*	KY394919	KY393513	*Primulinawentsaii*	KY395033	KY393592
*Primulinalinearicalyx*	MH032854	MH032841	*Primulinawuae*	MK747159	MK746265
*Primulinalinearifolia*	KY394921	KY393515	*Primulinaxiziae*	KY395038	KY393597
*Primulinalonggangensis*	JX506916	JX506808	*Primulinayangchunensis*	KY395039	KY393598
*Primulinalongicalyx*	KY394927	KY393521	*Primulinayangshanensis*	KY395040	KY393599
*Primulinalongii*	JX506917	JX506809	*Primulinayangshuoensis*	KY395042	KY393601
*Primulinalongnanensis*	OP243286	OP243282	*Primulinayingdeensis*	KU528876	KU528886
*Primulinalongzhouensis*	JX506918	JX506810	*Primulinayungfuensis*	JX506957	JX506848
*Primulinalunglinensis*	KY394930	KY393524	*Primulinazhoui*	MK747104	MK746222

### ﻿Phylogenetic analyses

We constructed Maximum Likelihood (ML) and Bayesian Inference (BI) phylogenetic trees for *Primulinananlingensis* and 124 *Primulina* species using ITS and *trn*L-F sequences with PhyloSuite v.1.2.2 (Zhang et al. 2020). Sequences were aligned using MAFFT v.7.471 (Katoh et al. 2019) in PhyloSuite v.1.2.3 and refined with Gblocks 0.91b (Talavera and Castresana 2007), prior to their combination. The optimal nucleotide substitution model was determined using ModelFinder (Kalyaanamoorthy et al. 2017) in PhyloSuite v.1.2.3. The ML tree was built using IQ-tree v.1.6.12 (Nguyen et al. 2015) with the TN+F+R3 model and 5000 bootstrap replicates. The BI tree was constructed using MrBayes v.3.2.6 (Ronquist et al. 2012) with the HKY+F+I+G4 model, running two chains for 3,000,000 generations and sampling every 1000 generations. Both models were selected, based on the Bayesian Information Criterion (BIC). Finally, tree visualisation was performed using ITOL v.7 (https://itol.embl.de/).

## ﻿Result

### ﻿Molecular phylogenetic studies

**Figure 1.**
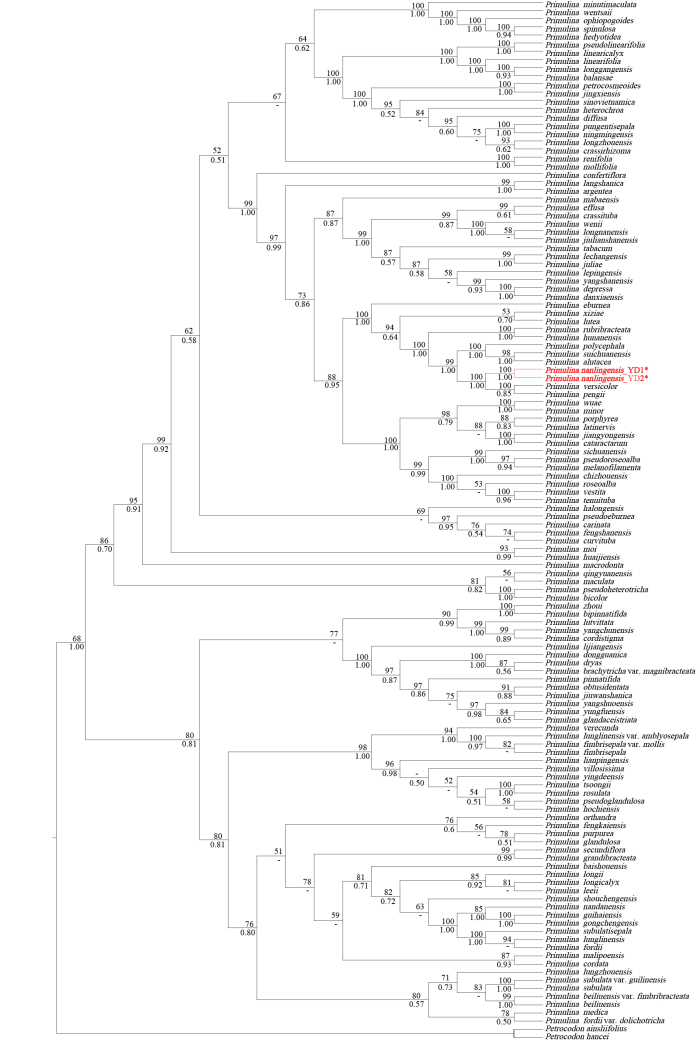
Phylogenetic tree of *Primulina* generated using Maximum Likelihood (ML) analysis and Bayesian Inference (BI) of the combined ITS and *trn*L-F sequences. Numbers on branches indicate bootstrap support (≥ 50%) from ML and posterior probabilities (≥ 0.50, rounded to two decimal places) from Bayesian Inference (BI) analyses, while values (< 50% / 0.50) below this threshold are represented by a dash (–). * indicates the new species.

The ITS matrix, consisting of 128 sequences with an aligned length of 952 base pairs (bp), contained 52.5% variable sites and 34.5% informative sites. Similarly, the *trn*L-F matrix had an aligned length of 1027 bp, with 20.8% variable sites and 9.2% informative sites. Additionally, the combined ITS and *trn*L-F matrix had a total aligned length of 1689 bp and featured 38.8% variable sites and 24% informative sites. The two populations of the new species from Shimentai National Nature Reserve form a monophyletic group (BS = 100%, PP = 1.00), where BS stands for bootstrap support and PP stands for posterior probability. They also form a sister clade with *P.versicolor* and *P.pengii* (BS = 100%, PP = 1.00). All three form a strongly-supported clade with *P.alutacea*, *P.suichuanensis* and *P.polycephala* (Fig. 1).

### ﻿Taxonomic treatment

#### ﻿ Primulina nanlingensis

Taxon classificationPlantaeLamialesGesneriaceae

J.C.Luo & H.F.Chen sp. nov.

D9AE6CC0-7575-5C1A-B2E7-5B2890B0BAFA

urn:lsid:ipni.org:names:77359322-1

Figs 2 , 3

##### Type.

China • Guangdong Province, Yingde City, Shimentai National Nature Reserve, 23°28′N, 113°05′E, 620 m elev., growing on top of a cliff on a limestone hill, 7 May 2024 (fl.), *J.C. Luo & H.F. Chen LJC00501* (holotype: IBSC; isotypes: IBSC).

**Figure 2.**
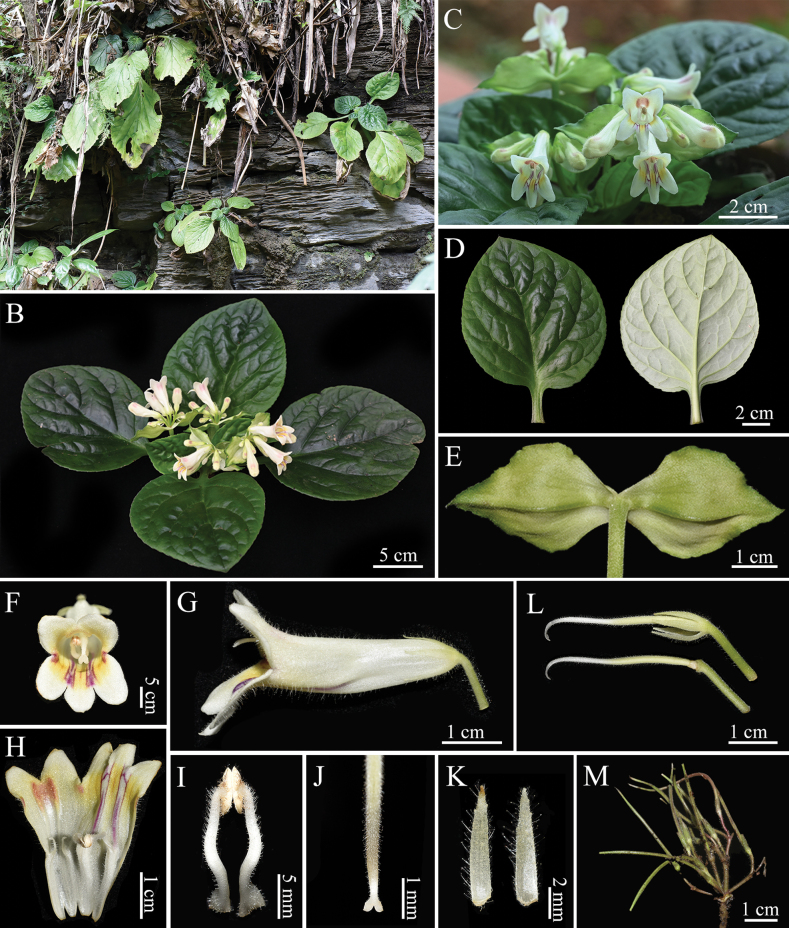
*Primulinananlingensis* J.C.Luo & H.F.Chen **A** plants in natural habitat **B** habit in flowering **C** cyme and frontal view of corolla **D** the adaxial and abaxial surface of leaf blades **E** outside surface of bracts **F** frontal view of corolla **G** side view of corolla **H** opened corolla showing stamens, staminodes and colour **I** stamens **J** stigma **K** outside and inside surface of calyx lobes **L** pistil with calyx lobes and pistil without calyx lobes **M** infructescence.

##### Diagnosis.

The new species is similar to *Primulinaversicolor* F.Wen, B.Pan & B.M.Wang in terms of flower shape and corolla colour, but easily distinguished from the larger leaf blades (10–21 × 7–19 cm vs. 8–18 × 6.5–16.5 cm) with a crenate margin (vs. entire); notably lower number of flowers (3–4 cymes, 4–8 flowered vs. 4–8 cymes, 4–24 flowered or more); bracts ovate-lanceolate (vs. broadly oval or suborbicular), with shallowly serrate margins above the middle (vs. entire margins); calyx lobes densely glandular on both surfaces (vs. outside glandular-pubescent inside nearly glabrous) and with 1–3 inconspicuous teeth each side (vs. 3–5-serrate); longer pistil (3.2–3.5 cm vs. 2.5–2.8 cm) and glandular-puberulent (vs. puberulent); filaments white (vs. pale yellow) with densely glandular at base and tip, sparser mid-section (vs. only upper half sparsely glandular-puberulent). Additionally, while the leaf morphology of this new species resembles that of *P.pengii* W.B.Xu & K.F.Chung, it differs in having a longer corolla length (4.2–5.2 cm vs. 2.8–3.6 cm), pale yellow corollas (vs. white) and ovate-lanceolate bracts with slightly serrate edges above the middle (vs. cordate bracts with entire margins).

**Figure 3.**
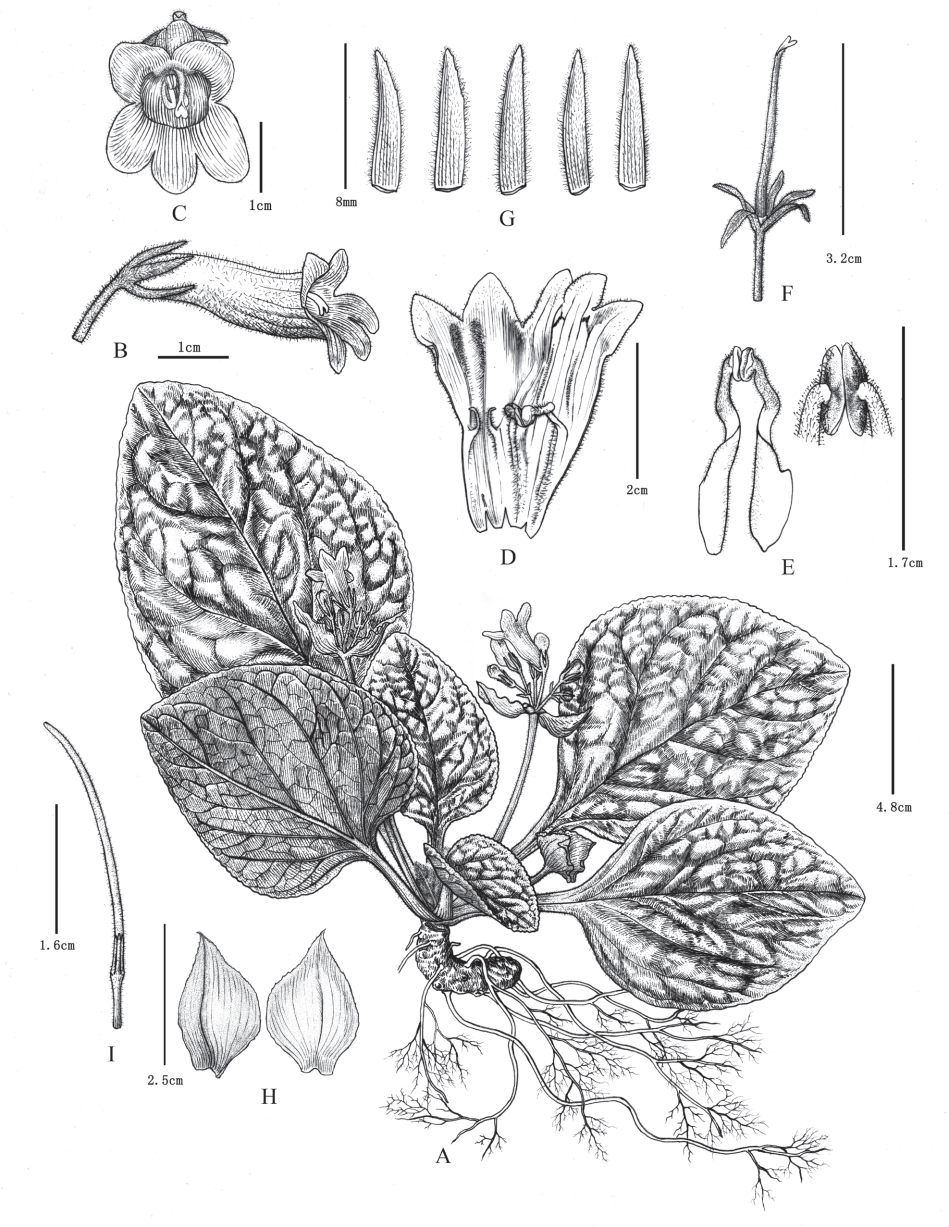
*Primulinananlingensis* J.C.Luo & H.F.Chen **A** habit **B** side view of corolla **C** frontal view of corolla **D** opened corolla **E** stamens **F** pistil with calyx lobes **G** outside surface of calyx lobes **H** outside surface of bracts **I** capsule. Drawn by Mrs. Yunxiao Liu based on *J.C. Luo & H.F. Chen LJC00501*.

##### Description.

Herbs, perennial rhizome subterete, 5–8 cm × 1.5–2.5 cm, internodes indistinct. Leaves 6–8, opposite at top of rhizome; blade green, succulent to thickly chartaceous, ovate or broadly ovate to elliptic, 10–21 × 7–19 cm, apex obtuse or subacute, margin crenate, base slightly oblique or symmetrical, leaf surface and petiole densely pubescent, abaxial surface densely villous and the veins on the abaxial surface densely pubescent; lateral veins 4–6-nerved on each side; petiole cross section sub-semicircular or compressed, 2.5–9.5 cm × 0.8–1.5 cm. Cymes axillary, 3–4, 4–8 flowers per cyme; bracts 2 opposite, ovate-lanceolate, 4.1–4.4 cm × 2.2–2.4 cm, outer side is shallowly serrate, apex acuminate, outside pubescent, inside sparsely pubescent; peduncle 5–12.5 cm long, 4–6 mm across, densely pubescent; pedicel 1.2–2.8 cm long, glandular-puberulent. Calyx 5-lobed nearly to the base, 8–15 mm × 1.6–2.5 mm, lanceolate, light green, both surfaces densely glandular-puberulent, with 1–3 inconspicuous teeth on each side. Corolla 4.2–5.2 cm long, pale yellow, throat dark yellow with 2 pale purple stripes, with 3 patches at the sinuses of the 2 upper lip lobes, patches light brown outside and dark purple inside, sometimes the dark purple patches absent and these patches are glandular, outside densely glandular-pubescent; tube infundibuliform, 3.2–3.8 cm long, orifice ca. 1.3 cm in diameter; limb distinctly 2-lipped, adaxial lip 2-lobed bifid to over the middle, lobes oblong, 5–7 mm × ca. 6 mm; abaxial lip 3-lobed to one-third from the top, lobes oblong, 4–6 mm × ca. 4 mm. Stamens 2, adnate to 1.3–1.5 cm above the base of the corolla base; anthers elliptic, 2.5–3 mm long, densely glandular; filaments ca. 15 mm long, white, with the base and upper part sparsely covered with glandular-puberulent; staminodes 3, two lateral ones adnate to ca. 1.3 cm above the corolla base, ca. 7 mm long, densely glandular-pubescent, apex bent, the central one adnate to ca. 3 mm above the base of corolla base, ca. 2 mm long, glabrous; disc annular, ca. 1 mm high, yellow, glabrous. Pistil 3.2–3.5 cm long; ovary linear, ca. 1.8–2.2 cm long, densely glandular-puberulent; style 0.8–1.2 cm long, sparse glandular-puberulent. Stigmas 2-lobed, 2.8–3.6 mm long, shallowly lobed, lobes ca. 1 mm long. Capsule green, mature dark brown, 3.2–4.5 cm × 1.5–2.2 mm, with persistent calyx lobes at base, densely white-villous and pubescent.

##### Phenology.

Flowering from late April to early June, fruiting from June to August.

##### Distribution and ecology.

*Primulinananlingensis* is known only from two separate limestone hills in the Shimentai National Nature Reserve, Yingde City, Guangdong Province, China. Companion species were calcareous herbs such as *Selaginellaeffusa* Alston, *S.delicatula* (Desv.) Alston., *Pileapeltata* Hance and Ficussarmentosavar.henryi (King ex Oliv.) Corner etc.

##### Etymology.

The species epithet refers to the type locality, the Nanling Mountains.

##### Vernacular name.

南岭报春苣苔 (Chinese name); Nán Lǐng Bào Chūn Jù Tái (Chinese pronunciation).

##### Provisional conservation status.

At present, only two populations of *Primulinananlingensis* have been discovered on limestone hills in the Shimentai National Nature Reserve, where a substantial area of 35 km^2^ has been identified as suitable habitat for the species. The two naturally distributed populations are no more than 10 km apart and each population consists of no more than 100 mature individuals. Currently, the two populations are stable, as the habitat is under protection by the administrators of the scenic area. However, considering the overall low number of individuals across the species populations and the conservation measures in place, it could be provisionally classified as Near Threatened [NT] according to the IUCN Red List Categories and Criteria (IUCN Standards and Petitions Committee 2024).

##### Additional specimens examined.

China • Guangdong Province, Yingde City, Shimentai National Nature Reserve, 23°28′N, 113°05′E, 620 m elev., growing on top of a cliff on a limestone hill, 25 May 2024 (fl.), *J.C. Luo & H.F. Chen LJC00502* (IBSC).

## ﻿Discussion

**Figure 4.**
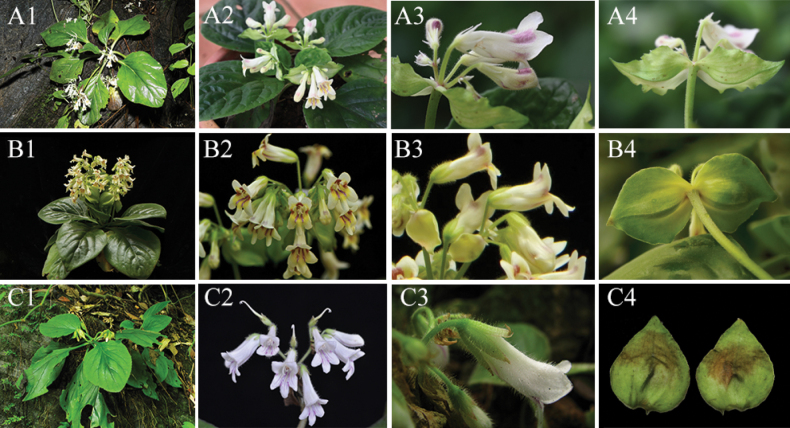
Morphological comparison between *Primulinananlingensis* (**A1–A4**), *P.versicolor* (**B1–B4**, photos by Fang Wen.) and *P.pengii* (**C1–C4**, photos by Weibin Xu). Legends: Leaf blade (**A1, B1, C1**); Cyme (**A2, B2, C2**); Corolla and Calyx lobes (**A3, B3, C3**); Bracts (**A4, B4, C4**).

The karst regions of southern and south-western China, as well as northern Vietnam, are hotspots for diversity of *Primulina* species, predominantly consisting of endemic species with limited populations confined to isolated sites (Kang et al. 2014; Tong et al. 2020; Wei et al. 2022). The Nanling Mountain Range serves as a habitat for *Primulina*, where the complex topography and soil heterogeneity foster a high level of species diversity and endemism (Wang et al. 2017). The type localities for both *P.nanlingensis* and *P.versicolor* are situated within Yingde City, with a distance of over 50 km between them. Furthermore, *P.pengii* is found in Yangshan County, Guangdong Province, which is more than 120 km away from *P.nanlingensis*. Notably, *P.nanlingensis* closely resembles *P.versicolor* in both flower shape and colour, but differs in several internal floral structures, such as the pistil with glandular-pubescent and stamens densely glandular at the base and tip, sparser in the middle, fewer flowers per cyme and overall cyme number. Phylogenetic analysis shows that they are closely related, yet their morphological differences suggest that *P.nanlingensis* represents a new species. Detailed comparisons of the three species are provided in Table 2 and Fig. 4. Finally, given the small population size and restricted distribution to just two locations, conservation efforts for *P.nanlingensis* are of utmost importance. In all, *P.nanlingensis*, as a newly-discovered species in *Primulina*, not only enhances the plant diversity in Naning Mountain, but also provides valuable insights for further study on the local adaptation in karst regions.

**Table 2.** Comparisons of *Primulinananlingensis* to *P.versicolor* and *P.pengii*, respectively.

Part	*P.nanlingensis*	*P.versicolor*	*P.pengii*
Leaf blade	ovate or broadly ovate to elliptic, 10–21 cm × 7–19 cm, margin crenate	broadly oval or nearly cordate, 8–18 cm × 6.5–16.5 cm, margin entire	ovate to broadly ovate, 14–25 cm × 9.5–15 cm, the margin shallowly repand to crenate
Cyme	3–4, 4–8-flowered	4–8, 4–24-flowered or more	3–4, 4–12-flowered
Bracts	ovate-lanceolate, 4.1–4.4 cm × 2.2–2.4 cm, shallowly serrate above the middle, apex acuminate, outside pubescent, inside sparsely pubescent	broadly oval or suborbicular, 5–5.5 cm × 4.4–5 cm, apex acute, outside densely appressed pubescent, inside nearly glabrous, margin entire	cordate, 2.6–3.2 cm × 2.5–3 cm, the margin entire to shallowly repand, the apex acute, outside pubescent, inside sparsely pubescent
Corolla	pale yellow, 4.2–5.2 cm, outside densely glandular-pubescent, inside nearly glabrous; throat dark yellow with 2 pale purple stripes	canary yellow, 3.5–4.2 cm, outside densely glandular-pubescent, inside nearly glabrous; throat dark yellow with 2 brownish-purple stripes	white, 2.8–3.6 cm long, outside glandular pubescent, inside sparsely puberulent, with 2 pale purple stripes
Calyx lobes	8–15 mm × 1.6–2.5 mm, both surfaces densely glandular, with 1–3 inconspicuous teeth each side	8.5 mm × 2 mm, outside densely glandular-pubescent, inside nearly glabrous, margin 3–5-serrate	8–10 mm × ca. 2 mm, outside glandular pubescent, inside sparsely pubescent, margin serrulate
Stamens	filaments ca. 15 mm long, white, densely glandular at base and tip, sparser mid-section, anthers elliptic, 2.5–3 mm long, densely glandular	filaments ca. 12.5 mm long, pale yellow, glabrous, but the upper half of filament sparsely glandular puberulent, anthers semicircular, 5–6 mm long, glabrous	filaments ca. 14 mm long, white, sparsely puberulent, anthers reniform, ca.4 mm long, puberulent
Pistul	3.2–3.5 cm long, densely glandular-puberulent	2.5–2.8 cm long, densely puberulent	2.4–3.1 cm long, densely puberulent

## Supplementary Material

XML Treatment for Primulina nanlingensis

## ﻿Additional information

### Author ORCIDs

Jin-Chu Luo https://orcid.org/0009-0003-0526-8962

Yuan-Qiu Li https://orcid.org/0009-0005-8135-500X

Ya-Li Li https://orcid.org/0000-0003-4667-5241

Ming-Zhao She https://orcid.org/0009-0003-1223-6037

Fa-Guo Wang https://orcid.org/0000-0002-9326-8000

Hong-Feng Chen https://orcid.org/0000-0002-8415-3260

### Data availability

All of the data that support the findings of this study are available in the main text.
